# Creating a Team to Focus on Diversity and Inclusion in Research Education and Recruiting

**DOI:** 10.1089/heq.2023.0018

**Published:** 2023-05-26

**Authors:** Claudia Gutierrez, Jennifer Deen, Lori Churby, Rachel Ramoni, Molly Klote

**Affiliations:** ^1^ Million Veteran Program Coordinating Center, VA Palo Alto Health Care System, Palo Alto, CA.; ^2^ Million Veteran Program Office, Office of Research and Development, Department of Veterans Affairs, Washington, District of Columbia, USA.; ^3^ Chief Research and Development Officer, Office of Research and Development, Department of Veterans Affairs, Washington, District of Columbia, USA.; ^4^ Deputy Chief Research and Development Officer Enterprise Support, Office of Research and Development, Department of Veterans Affairs, Washington, District of Columbia, USA.

In early summer 2020, during the outbreak of the SARS-CoV-2 (COVID-19) pandemic, the United States federal research response to COVID-19 was underway. The White House invited the Department of Veterans Affairs (VA) to participate in the federal response to quickly develop and test safe and effective vaccine candidates and treatments. VA was ideally suited to support this effort as the largest integrated health care system in the United States. Over six million patients are actively cared for by VA, and >100 of VA's medical centers conduct research.

In total, VA supported a wide range of research efforts that included four industry-sponsored COVID-19 vaccine trials conducted at 20 VA medical centers; therapeutic treatment trials under the National Institutes of Health Accelerating COVID-19 Therapeutic Interventions and Vaccines (ACTIV) portfolio; a study site for the nationwide Researching COVID-19 to Enhance Recovery (RECOVER) initiative, a large-scale study on the long-term effects of COVID-19; and a therapeutic intervention using a prostate cancer drug to target the COVID-19 virus in the lungs.

These research efforts were fast-paced, and the pressures of the pandemic required swift action to recruit diverse participants into the trials. Yet one of the most significant barriers to successfully launching a research study is the ability to recruit enough participants. Even more challenging is the recruitment of participants from diverse racial and ethnic backgrounds: people who are historically under-represented in medical research.

Many factors lead people to seek out and enroll in a study, and just as many factors contribute to lack of participation, ranging from a lack of awareness to outright fear of participation in medical research. Some of these concerns are rooted in past mistreatment of under-represented groups, as exemplified by the 1932 Tuskegee study of untreated syphilis in Black men, who were left untreated for their disease, even after therapy became available.

For the health and safety of veterans and others, it was deemed essential that studies of vaccines and treatments for COVID-19 reflected the diversity of the U.S. population. Furthermore, it was important to over-index Black and Hispanic communities, as well as women and other racial and ethnic minorities, who suffered the highest morbidity and mortality rates from COVID-19. Inclusion of these groups in COVID-19 research helped ensure that new vaccines and treatments were safe and effective in people of all backgrounds. In addition, communities that participated in medical research were more likely to accept medical interventions that had been tested in studies that included people from similar backgrounds and races.

To encourage diverse participation in VA COVID-19 research, recruitment materials transparently described the risks and benefits of research studies in plain language. For many individuals, the materials were their first interface with COVID-19 research at VA. The messaging was culturally sensitive and written to promote trust and convey diverse recruitment goals. In addition, the VA Office of Research and Development (ORD) established the Diversity and Inclusion (D&I) subcommittee of the National Research Advisory Committee (NRAC) to help accomplish this task.

ORD recruited D&I subcommittee members who had expertise in communicating to diverse audiences. Despite busy schedules and additional COVID-19 workloads, these individuals were eager to help. Representatives from the following offices were contacted and agreed to participate:

National Veterans Outreach Groups
Center for Minority Veterans
National Center for Homelessness Among Veterans
Center for Development and Civic Engagement (formerly Voluntary Services)
Ambulatory Care
Medical Media and External Affairs
VISN 9 Communications Officer
Social Worker from Hampton VAMC
Women's Health Research Network
Office of Community Engagement
Office of Intergovernmental Affairs
Office of National Chaplain Services
Office of Public and Intergovernmental Affairs
U.S. Digital Services

With 12 voting members, the task of reviewing communications strategies, materials, and informed consent documents began at the first meeting in October 2020. Of primary focus were materials and approaches for recruiting diverse audiences into the newly established VA Volunteer Registry for COVID-19 Research. Individuals who signed up for the registry agreed to be contacted about VA COVID-19 clinical trials in their area. If volunteers met eligibility requirements and confirmed continued interest in participating in research, their information was shared with research teams. Once contacted by the study team, volunteers had the opportunity to accept or decline participation in a study, and complete informed consent materials. This process also allowed for oversampling of under-represented populations.

The D&I subcommittee advised researchers to use a multichannel approach that included print and digital media, including a communication toolkit, geotargeted mailings and email campaigns, a 30-sec public service announcement, media engagement, social media, human-interest stories posted to VA's main news website, and feature stories posted to participating VA medical center websites. Committee members ensured messaging and distribution tactics were culturally sensitive and appropriate.

The subcommittee also had a synergistic effect in enabling and promoting outreach. Outside of VA, members of the committee also belonged to other organizations, such as sororities and fraternities, and leveraged their connections to activate those networks. Outreach extended to faith communities and veterans in rural America. The subcommittee was very effective in educating researchers and ORD leadership on selecting the best messenger for each community.

Although the subcommittee's work began with COVID-19 research, it has since extended its efforts. The most recent success was in advising the VA Million Veteran Program (MVP) during the design of its first-ever large-scale digital outreach campaign, which was also the first campaign to specifically recruit women veterans into MVP. One committee member volunteered to help MVP organize, advertise, and host a Facebook Live event that vastly expanded campaign reach. Other committee members distributed campaign materials among women veterans within their spheres of influence, helping amplify the spread and impact of campaign messaging among women veteran communities.

There is no shortage of opportunities for the subcommittee to enhance VA research recruitment efforts, thereby ensuring that VA has the most diverse and representative research in the country. Organizations that want diverse and inclusive medical research participation might consider an approach that involves a D&I review committee.

## Abbreviations Used

D&I: Diversity and Inclusion
MVP: Million Veteran Program
ORD: Office of Research and Development
VA: Department of Veterans Affairs

